# 

**Published:** 1919-12

**Authors:** 


### The Library of the Bristol Medico-Chirurgical Society.

*October 31st*, 1919.

*The following donations have been received since the publication of the list in the Spring Number*, 1919.

The figures in brackets refer to the figures after the names of the donors, and show by whom the volumes were presented. The books to which no such figures are attached have either been bought from the Library Fund or received through the *Journal*.

| | | |
|---|---|---|
| Lady Baron (1) .. .. .. .. .. | 78 | volumes |
| Mrs Bullen (2) .. .. .. .. .. | 53 | „ |
| Mrs. Michell Clarke (3) .. .. .. .. | 109 | „ |
| Dr. Carey Coombs (4) .. .. .. .. | 3 | „ |
| Dr. C. Elliott (5) .. .. .. .. .. | 56 | „ |
| Dr. J. M. Fortescue-Brickdale (6) .. .. | 2 | „ |
| Mr. L. M. Griffiths (7) .. .. .. .. | 2 | „ |
| Mr. E. W. Hey Groves (8) .. .. .. | 6 | „ |
| Mr. C. A. Joll (9) .. .. .. .. .. | 1 | volume. |

### NINETY-EIGHTH LIST OF BOOKS.

| | | | |
|---|---|---|---|
| **Aitken, W.** .. .. | *Science and Practice of Medicine.* 2 vols. | (5) 6th Ed. | 1872 |
| **Allen, R. W.** .. .. | *Practical Vaccine Treatment* .. .. .. .. | | 1919 |
| **Althaus, J.** .. .. | *On Epilepsy, Hysteria and Ataxy* .. .. | (5) | 1866 |
| **Anderson, M'C.** .. | *Parasitic Affections of the Skin* | (5) 2nd Ed. | 1868 |
| **Archbold,** [—] .. | *Lunacy* (Ed. by G. S. Lushington) | (2) 4th Ed. | 1895 |
| **Ash, E. L.** .. .. | *How to Treat by Suggestion* .. .. .. | (2) | [1914] |
| **Barrett, J. W.** .. | *A Vision of the Possible* .. .. .. .. .. .. | | 1919 |
| **Bartholow, R.** .. | *Materia Medica* .. .. .. | (1) 4th Ed. | 1882 |
| **Basu, B. D.** .. .. | *Diabetes and its Dietetic Treatment* .. | 10th Ed. | 1919 |
| **Berkeley, C.** (Ed.) .. | *Midwifery* .. .. .. .. .. .. .. .. .. .. | | 1917 |
| „ .. | *Diseases of Women* .. .. .. .. .. .. .. .. | | 1919 |
| **Beaupré, M.** .. .. | *Effects and Properties of Cold* .. .. .. | (5) | 1826 |
| **Bell, W. B.** .. .. | *The Pituitary* .. .. .. .. .. .. .. .. .. | | 1919 |
| **Bayliss, W. M.** .. | *An Introduction to General Physiology* .. .. | | 1919 |
| **Blandford, G. F.** .. | *Insanity* .. .. .. .. .. .. | (2) 2nd Ed. | 1877 |
| **Blake, E. T.** .. .. | *Septic Intoxications* .. .. .. .. .. .. | (2) | [1892] |
| **Borradaile, L. A.** .. | *Elementary Zoology* .. .. .. .. | 2nd Ed. | 1918 |
| **Bowlby, A. A.** .. | *Surgical Pathology* .. .. .. .. .. | 4th Ed. | 1900 |

| | | | | |
|---|---|---|---|---|
| **Bradby, K. M.** | *Psycho-Analysis* | | | 1919 |
| **Browne, L.** | *Voice, Song and Speech* | (1) | 3rd Ed. | 1884 |
| ,, | *Voice : Use and Stimulants* | (1) | | 1885 |
| **Buschan, G.** | *Menschenkunde* | (1) | | [1909] |
| **Buzzard, T.** | *Diseases of the Nervous System* | (1) | | 1882 |
| **Byam, W.** [*et al*] | *Trench Fever* | | | 1919 |
| **Canney, L.** | *Meteorology in Egypt* | (1) | | 1897 |
| **Carrell, and R. M. Wilson, J. H.** | *The Nervous Heart* | | | 1919 |
| *Catalogue of Lewis's Medical and Scientific Library* | | | | 1918 |
| **Celsus, A. C.** | *De Medicinæ Libri Octo* (Trans. by Lee) | (5) | | 1831 |
| **Chandler, A. C.** | *Animal Parasites and Human Disease* | | | 1918 |
| **Cole, S. W.** | *Practical Physiological Chemistry* | | 5th Ed. | 1919 |
| **Crocker, H. R.** | *Atlas of Skin Diseases.* 2 vols. | | | 1903 |
| **Cunning, and C. A. Joll, J.** | *Aids to Surgery* | (9) | 4th Ed. | 1919 |
| **Day, W. H.** | *Headaches* | (2) | 3rd Ed. | 1880 |
| **Deane, H. E.** | *Gymnastic Treatment for Joint Disabilities* | | | 1918 |
| **Dexter, and A. H. Garlick, T. F. G.** | *Psychology in the Schoolroom* | (2) | | 1908 |
| **De Schweinitz, G. E.** | *Diseases of the Eye, Nose and Throat* | (1) | Vol. II. | 1899 |
| *Dictionary of National Biography. Index and Epitome* | | (3) | | 1903 |
| **Douthwaite, A. W.** | *Fevers in China* | (5) | | 1892 |
| **Duval, P.** | *War Wounds of the Lung* | | | 1918 |
| **Eassie, W.** | *Healthy Homes* | (5) | 2nd Ed. | 1876 |
| **Effront, J.** | *Biochemical Catalysts* (Tr. by S. C. Prescott) | | | 1917 |
| **Ferrier, D.** | *The Harveian Oration* | | | 1902 |
| **Field, G. P.** | *Diseases of the Ear* | (1) | 4th Ed. | 1893 |
| ,, | *Suppurative Diseases of the Ear* | (1) | | 1893 |
| **Garrod, A.** | *Materia Medica* | (1) | 7th Ed. | 1879 |
| **Gibbes, H.** | *Practical Histology* | (5) | 2nd Ed. | 1883 |
| **Giles, A. E.** | *Sterility in Women* | | | 1919 |
| **Goodall, E.** | *Microscopical Anatomy of the Human Brain* | (2) | | 1894 |
| ,, | *The Croonian Lectures* | (2) | | 1914 |
| **Guenther, K.** | *Der Kampf um das Weib* | (1) | | 1909 |
| **Guy, and D. Ferrier, W. A.** | *Forensic Medicine* | | 6th Ed. | 1888 |
| **Halliburton, W. D.** | *Handbook of Physiology* | | 14th Ed. | 1919 |
| **Hammond, R.** | *Diseases of the Nervous System* | (1) | 7th Ed. | 1881 |
| **Harrison, R.** | *Dublin Dissector.* Vol. I. | (5) | 5th Ed. | 1847 |
| **Hirschell, G.** | *Local Anæsthesia* (Tr. by R. E. Krohn) | | | 1914 |
| **Hilton, J.** | *Rest and Pain* (Ed. by W. Jacobson) | (1) | 4th Ed. | 1887 |
| **Hudson, A.** | *Lectures on Fever* | (5) | 2nd Ed. | 1868 |
| **Hurst, A. F.** | *Constipation and Allied Intestinal Disorders* | | 2nd Ed. | 1919 |
| **Jackson, R.** | *Effects of Cold Water* | (5) | | 1808 |
| **Joal, [—]** | *De la Respiration dans le Chant* | (1) | | [N.D.] |
| **Joll, J. Cunning and C. A.** | *Aids to Surgery* | (9) | 4th Ed. | 1919 |
| **Kaposi, M.** | *Pathologie und Therapie der Syphilis.* Part I. | (1) | | 1881 |
| **Keith, A.** | *Menders of the Maimed* | | | 1919 |

**Kraepelin, E.** .. *Dementia Præcox* (Tr. by R. M. Barclay, Ed. by G. M. Robertson) .. .. .. .. .. 1919
**Lang, W. D.**.. .. *Distribution of Mosquitoes in England and Wales* 1918
**Latham, and C. H. Garland, A.** *The Conquest of Consumption* .. 1911
**Lawrence, W.** .. *Diseases of the Eye* .. .. .. .. .. 3rd Ed. 1844
**Lawrie, J. M.** .. *A War Cookery Book for the Sick* .. 6th Ed. [N.D.]
*Liverpool Public Libraries, Handbook of* .. .. .. .. .. .. .. .. 1912
*Liverpool, City of : Official Handbook* .. .. .. .. .. .. .. .. .. 1912
**Love, J. K.** .. .. *Ear Disease in School Children* .. .. .. .. 1919
**McBride, P.** .. .. *Diseases of Throat, Nose and Ear* (1) 2nd Ed. 1894
**Mackenzie, Sir J.** .. *The Future of Medicine* .. .. .. .. .. .. 1919
**Makins, G. H.** .. *Gunshot Injuries to the Blood-Vessels* .. .. 1919
**Marr, H. C.** .. .. *Psychoses of the War*.. .. .. .. .. .. .. 1919
**Martin, J. M. H.** .. *Illustrated Ambulance Lectures* .. 3rd Ed. 1892
**Marshall, J.** .. .. *Outlines of Physiology.* 2 vols. .. .. .. .. 1867
**Mitchell, S. W.** .. *Diseases of the Nervous System* .. .. .. 1881
**Moure, E. J.** .. .. *Pathologie de Guerre du Larynx et de Trachée*.. 1918
**Monrad-Krohn, G. H.** *Om Abdominal-Reflexerne* .. .. .. .. 1918
**Morgan, C. L.** .. *Eugenics and Environment* .. .. .. .. 1919
*Nomenclature of Diseases* .. .. .. .. .. .. .. .. (1) 5th Ed. 1918
**Oppert, F.** .. .. *Hospitals, Infirmaries and Dispensaries* 2nd Ed. 1883
*Pamphlets.* 6 vols. .. .. .. .. .. .. .. .. .. .. .. .. (2) [V.Y.]
**Park, W. H.** .. .. *Opium in China* .. .. .. .. .. .. (5) 1899
**Pear, G. E. Smith and T. H.** *Shell Shock and its Lessons* .. .. .. 1919
**Politzer, A.** .. .. *Diseases of the Ear* (Trans. by J. P. Cassells) (1) 1883
**Powell, Sir R. D.** .. *The Harveian Oration* .. .. .. .. .. .. 1914
**Rees, F.** .. .. .. *National Health* .. .. .. .. .. .. .. .. 1919
**Reid, and W. E. Hicks, W. N.** *Leading Events in the History of Bristol* .. .. .. .. .. .. .. .. (7) [N.D.]
**Riviere, C.** .. .. *The Early Diagnosis of Tubercle* .. 2nd Ed. 1919
**Rogers, Sir L.** .. *Fevers in the Tropics* .. .. .. 3rd Ed. 1919
**Shera, A. G.** .. .. *Vaccines and Sera* .. .. .. .. .. .. .. .. 1918
**Smith, and T. H. Pear, G. E.** *Shell Shock and its Lessons*.. .. .. 1919
**Stewart, T. G.** .. *Bright's Disease* .. .. .. (1) 2nd Ed. 1871
**Stierlen, E.** .. .. *Klinische Röntgendiagnostik des Verdauungskanals* .. .. .. .. .. .. .. .. .. 1916
**Sutton, H. G.**.. .. *Medical Pathology* .. .. .. .. .. .. (1) 1886
**Tanner, T. H.** .. *Manual of Clinical Medicine* .. (5) 2nd Ed. 1869
" .. .. *Diseases of Infancy* .. .. .. (1) 3rd Ed. 1879
**Thomson, Sir St. C.** *John C. Lettsom and the Foundation of the Medical Society* .. .. .. .. .. .. .. 1918
**Tinel, J.** .. .. .. *Nerve Wounds* (Ed. by C. A. Joll) .. .. .. 1917
*Trench Fever. Medical Research Committee* .. .. .. .. .. .. 1918
**Tuke, D. H.** .. .. *Influence of the Mind upon the Body* .. (1) 1872
**Tweedy, and G. T. Wrench, E. H.** *Practical Obstetrics* .. 4th Ed. 1919
**Waggett, E. B.** .. *Diseases of the Nose* .. .. .. .. .. (1) 1907
**Walshe, W. H.** .. *Diseases of the Heart* .. .. (1) 4th Ed. 1873
**Waring, E. J.** .. *Practical Therapeutics* .. .. (5) 3rd Ed. 1871

**Wilson, and J. H. Carrell, R. M.** *The Nervous Heart* .. .. .. 1919
**Wrench, and E. H. Tweedy, G. T.** *Practical Obstetrics* .. 4th Ed. 1919
**Yearsley, J.** .. .. *Deafness* .. .. .. .. .. .. (5) 6th Ed. 1863
**Youatt, W.** .. .. *The Horse : Management and Diseases* (5) 1870

TRANSACTIONS, REPORTS, JOURNALS, ETC.

American Association of Obstetricians and Gynecologists, Transactions of the .. .. .. .. .. .. .. .. .. .. Vol. XXXI. 1918
American Journal of Surgery .. .. Vols. XXX.—XXXII. 1916–18
American Journal of the Medical Sciences
Vols. (3) CLI.—CLIII. 1916–17 ; (6) CLIV., CLV. 1917–18
American Laryngological, Rhinological and Otological Society, Transactions of the.. .. .. .. .. .. .. .. .. .. .. .. 1918
American Pediatric Society, Transactions of the .. Vol. XXX. 1918
Annales de Dermatologie .. .. .. .. .. .. .. .. .. .. .. 1915
British Journal of Dermatology .. .. Vols. XXVIII., XXIX. 1916–17
Defective and Epileptic Children, Report of the Committee on
(7) Vol. I. 1898
Hospitals and Charities, Burdett's .. .. .. .. .. .. .. .. .. 1918
International Abstract of Surgery.. ..(8) Vols. XXIII.—XXV. 1916–17
Johns Hopkins Hospital Bulletin, The Vols. XXVII., 1916 ; XXIX., 1918
Journal of Experimental Medicine.. .. Vols. XXIV.—XXVII. 1916–18
Journal of Laryngology, Rhinology and Otology
Vols. XXXI.—XXXIII. 1916–18
Journal of Medical Research .. .. Vols. XXXV.—XXXVIII. 1916–18
Library Association Record .. .. .. .. .. .. .. Vol. XVI. 1914
Library, The .. .. .. .. .. .. .. .. .. (N.S.) Vol. V. 1914
Medical Annual, The .. .. .. .. .. .. .. .. .. .. .. .. .. 1919
Medical Society's Transactions .. .. .. .. .. .. Vol. XLI. 1918
Quarterly Journal of Medicine .. .. .. (4) Vols. IX.—XI. 1915–18
Royal Society of Medicine, Proceedings of the Vols. IX.—XI. 1916–18
Studies from the Department of Pathology, Columbia University
Vol. XIV. 1914
Surgery, Gynecology and Obstetrics ..(8) Vols. XXIII.—XXV. 1916–17

### MEETINGS OF SOCIETIES.

### Bristol Medico-Chirurgical Society.

### Local Medical Notes.

### EXAMINATION SUCCESSES.

Students of the University have recently passed the following examinations :—

UNIVERSITY OF BRISTOL.—*M.B., Ch.B., First Examination, Part I. :* A. T. Binnie, S. H. Blacker, A. P. Bodman, N. Joan Bown, E. G. Bradbeer, E. R. Clutterbuck, Mary F. Dalton, E. Davis, B. Veronica F. Dawkins, H. Mary Dixon, R. H. Dummett, G. R. Dunn, F. R. Edbrooke, E. B. Eedle, N. J.

England, P. G. Evans, H. M. Golding, J. L. Griffin, A. G. Heron, J. A. James, F. G. Jenkins, Doris E. Joscelyne, P. C. Joscelyne, A. H. Marshall, J. R. Nicholson-Lailey, K. F. Platt, A. S. Prowse, H. Taylor, R. E. Whitby. *M.B., Ch.B., Part II.:* M. Critchley, J. R. Duerden, Phyllis T. Siepmann. *L.D.S., First Examination:* N. H. Bodenham, R. H. Davies, H. A. Jones. *L.D.S., Third Examination:* E. J. Tucker.

University of London.—*M.B., B.S., Third Examination:* G. M. Jackson, R. F. White. *Group II.:* J. A. Birrell.

F.R.C.S. Eng.—*Primary:* H. J. D. Smythe. *Final:* A. W. Adams, A. G. T. Fisher.

M.R.C.S., L.R.C.P.—B. A. Astley-Weston, G. T. Mody, G. A. Pennant, T. H. A. Pinniger, B. R. Reynolds, W. Worger, A. E. Young.

Royal College of Physicians, London.—Major F. P. Mackie, I.M.S., and H. Devine, M.D. Lond., elected Fellows.

Conjoint.—*Anatomy and Physiology:* Mary A. E. Somers. *Medicine:* F. S. Hunter, B. R. Reynolds. *Midwifery:* F. V. Jacques.

L.D.S., R.C.S. Eng.—L. H. Bradbeer, L. B. James.

L.S.A.—J. F. E. Burns, D. G. Cossham. *Forensic Medicine:* J. H. Eglinton.

Postgraduate Study.—The experience of the past year has shown that whilst a good attendance can be attained at postgraduate demonstrations during the months of May, June and July, it is difficult for busy practitioners to find the time during the autumn and early winter. It is proposed therefore for the 1920 session to give a course of about eight demonstrations during the summer months. The subjects will be announced later.

In the meantime if sufficient names are forwarded to the Director an effort will be made to extend the opportunities for postgraduate study in fresh branches of medical work, including tuberculosis, child welfare, venereal diseases and school inspection work. Medical men desirous of taking up postgraduate work during the winter months are reminded that clinical assistantships are open in all departments of medical and surgical work, and in the special departments. Where regular attendance is not possible, a man may attend any or every branch of hospital practice for a number of days equivalent to one month. The Director of Postgraduate Studies, Pathological Department, University of Bristol, will be pleased to answer any inquiries.

---

Illustrations.—*Contributors are asked to note that sketches for reproduction as line blocks should be drawn in black Indian ink on white paper, and of the actual size (quarter, half, or full page) they are to be reproduced.*

### INDEX.

Abdomen, The systematic examination of the—Dr. R. G. P. Lansdown, 33.
Abdominal surgery, Some developments in—F. Lace, 1.

Baron, Sir B. J., Obituary notice of, 90, 100.
Bell, Dr. J. H., The case of, 19, 89.
**Books, Reviews of—**
Barrett, Sir J.W.—A vision of the possible, 169
Basu, R. D.—Dietetic treatment of diabetes, 81.
Blood pictures—Dr. C. Price-Jones, 83.
Carrel and G. Dehelly, A.—Treatment of infected wounds, 86.
Dehelly, A. Carrel and G.—Treatment of infected wounds, 86.
Diabetes, Dietetic treatment of—R. D. Basu, 81.
Dream psychology—Dr. M. Nicoll, 84.
Eder, M. D.—War shock, 81.
Gay, Dr. F. P.—Typhoid fever, 166.
Harrison, L. W.—Venereal diseases, 168.
Heart, Diseases of the—Dr. F. W. Price, 167.
Hearts of man—Dr. R. M. Wilson, 170.
Hygiene & public health—Dr. C. Porter, 85.
Hysterical disorders of warfare—Dr. L. R. Yealland, 162.
Insanity—Dr. E. G. Younger, 83.
McCarrison, Dr. R.—The thyroid gland, 82.
Mackenzie, Sir J.—The future of medicine, 164.
Marr, Dr.H. C.—Psychoses of the war, 167.
May, Dr. O.—The prevention of venereal diseases, 84.
Medicine, The future of—Sir J. Mackenzie, 164.
Nicoll, Dr. M.—Dream psychology, 84.
Porter, Dr. C.—Hygiene and public health, 85.
Price, Dr. F. W.—Diseases of the heart, 167.
Price-Jones, Dr. C.—Blood pictures, 83.
Psychoses of the war—Dr. H. C. Marr, 167.
Sutton, Sir J. B.—Tumours, 82.
Thyroid gland, The—Dr. R. McCarrison, 82.
Trench fever, 85.
Tumours—Sir J. B. Sutton, 82.
Typhoid fever—Dr. F. P. Gay, 166.
Typhoid and para-typhoid fevers, Surgical aspects of—Dr. A. E. Webb-Johnson, 165.
Venereal diseases—L. W. Harrison, 168.
Venereal diseases, Prevention of—Dr. O. May, 84.

**Books, Reviews of** (*continued*)—
Vision of thepossible, A.—Sir J. W. Barrett, 169.
War Shock—M. D. Eder, 81.
Webb-Johnson, Dr. A. E.—Surgical aspects of typhoid and para-typhoid fevers, 165
Wilson, Dr. R. M.—The hearts of man, 170.
Wounds, Treatment of infected—A. Carrel and G. Dehelly, 86.
Yealland, Dr. L. R.—Hysterical disorders of warfare, 162.
Younger, Dr. E. G.—Insanity, 83.
Bristol Medico-Chirurgical Society, 100, 174.
Bristol Royal Infirmary, History of, 87.

Chemo-therapeutics, Modern—Dr. M. Nierenstein, 151.
Chest wounds, Late effects of, 158.
Clarke, Dr. J. M., Obituary notice of, 18, 23.
Contemporary medicine from the standpoint of pathology—Dr. I. W. Hall, 105.
Coombs, Dr. C.—Report on medicine, 151.

Dobson, N. C., Obituary notice of, 177.

Fortescue-Brickdale, Dr. J. M.—Postgraduate study, 48.
Fractures of the femur, Late results in, 156.
Fractures, Treatment of ununited—E. W. H. Groves, 132.

Gordon, Dr. R. G.—Stammering as it occurred in the war, 143.
Groves, E. W. H.—Treatment of ununited fractures, 132.
Gunshot injury of nerves—C. A. Morton, 55.

Hall, Dr. I. W.—Contemporary medicine from the standpoint of pathology, 105.
Honours, War, 91, 104.
Hospital amalgamation, 92.

Head wounds, Late results of, 158.

Joint wounds, Late results of, 158.

Lace, F.—Some developments in abdominal surgery, 1.
Lansdown, Dr. R. G. P.—The systematic examination of the abdomen, 33.
Library of the Bristol Medico-Chirurgical Society, 93, 171, 175.

Mackie, Dr. F. P.—Disease in Mesopotamia, 118.
Mesopotamia, Disease in—Dr. F. P. Mackie, 118.
Mole, H. F., Obituary notice of, 31.
Morton, C. A.—Gunshot injury of nerves, 55.

Nerves, Gunshot injury of—C. A. Morton, 55.
Nierenstein, Dr. M.—Some aspects of modern chemo-therapeutics, 151.

Obituary, 23, 100, 177.

Osler, Sir W., Obituary notice of 183.

Pathology, Contemporary medicine from the standpoint of—Dr. I. W. Hall, 105.
Postgraduate courses, 89, 102, 184.
Postgraduate study—Dr. J. M. Fortescue-Brickdale, 48.

Scars, Surgical treatment of, 157.
Southern General Hospital, Closing of the 2nd, 91.
Spinal cord wounds, Late results of, 160.
Stammering as it occurred in the war—Dr. R. G. Gordon, 143.

Tetanus, 155.
Thurston, H. C., Obituary notice of, 182.

War wounds, Surgical conference for the study of, 155.

533694

### *PUBLICATIONS RECEIVED.*

From Edward Arnold, London :
*Diseases of Women.* By Ten Teachers, under the direction of Comyns Berkeley, M.A., M.D. 1919. Price 30s. net.

From Baillière, Tindall and Cox, London :
*The Pituitary.* By W. Blair Bell. 1919. Price 30s. net.

From John Bale, Sons & Danielsson, Ltd., London :
*Babies in Peril : Or Mother and Infant Welfare Centres.* By Edith M. Bennett. 1919. Price 6d. net.
*Eugenics and Environment.* By Professor C. Lloyd Morgan, LL.D., D.Sc., F.R.S. 1919. Price 2s.

From Henry Frowde and Hodder & Stoughton, London :
*Practical Obstetrics.* By E. Hastings Tweedy and G. T. Wrench, M.D. Fourth Edition. 1919. Price 21s. net.
*The Early Diagnosis of Tubercle.* By Clive Riviere, M.D., F.R.C.P. Second Edition. 1919. Price 10s. 6d. net.
*Psycho-Analysis and its Place in Life.* By M. K. Bradby. 1919. Price 8s. 6d. net.
*Fevers in the Tropics.* By Sir Leonard Rogers, C.I.E., M.D., F.R.C.P. Third Edition. 1919. Price 30s. net.
*Sterility in Women.* By Arthur E. Giles, M.D., B.Sc. 1919. Price 10s. net.
*The Nervous Heart.* By R. M. Wilson, Capt. R.A.M.C., and John H. Carroll, Major, M.C., U.S.A. 1919. Price 6s. net.
*Trench Fever.* By Major W. Byam, R.A.M.C. : Captains J. H. Carroll, U.S.R., J. H. Churchill, R.A.M.C.(T.), Lyn Dimond R.A.M.C., V. E. Sorapure, R.A.M.C., R. M. Wilson, R.A.M.C., and Ll. Lloyd, R.A.M.C.(T.). 1919. Price 10s. 6d. net.
*Constipation and Allied Intestinal Disorders.* By Arthur F. Hurst, M.A., M.D., F.R.C.P. Second Edition. 1919. Price 16s. net.
*Psychoses of the War.* By H. C. Marr, Lieut.-Col., R.A.M.C. 1919. Price 16s. net.
*Menders of the Maimed.* By Arthur Keith, M.D., F.R.C.S., F.R.S. 1919. Price 16s. net.
*The Future of Medicine.* By Sir James Mackenzie, F.R.S., M.D. 1919.

From Harrison and Sons, London :
*John Coakley Lettsom and the Foundation of the Medical Society.* By Sir St. Clair Thomson. 1918. Price 2s. 6d. [Reprint.]

From H. K. Lewis & Co., Ltd., London :
*Catalogue of Lewis's Medical and Scientific Library.* New Edition. 1918. Price 12s. 6d. (6s. to Subscribers).
*A Vision of the Possible : What the R.A.M.C. might become.* By James W. Barrett, K.B.E., C.B., C.M.G., M.D. 1919. Price 9s. net.
*Practical Vaccine Treatment for the General Practitioner.* By R. W. Allen, M.A., M.D., B.S. 1919. Price 7s. 6d. net.

From E. & S. Livingstone, Edinburgh :
*Dementia Præcox and Paraphrenia.* By Professor Emil Kraepelin. Translated by R. Mary Barclay, M.A., M.B. Edited by George M. Robertson, M.D. 1919. Price 15s. net.

From Longmans, Green & Co., London :
*Shell Shock and its Lessons.* By Professor G. Elliot Smith, M.D., F.R.S., and T. H. Pear, M.A., B.Sc. New Edition. 1919. Price 1s. 6d. net.
*An Introduction to General Physiology.* By W. M. Bayliss, M.A., D.Sc., F.R.S. 1919. Price 7s. 6d. net.

From Macmillan & Co., Ltd., London :
*The Journal of Industrial Hygiene.* Vol. I., Nos. 1–4, May—August, 1919. Price 21s. per annum.

From The Panini Office, Bahadurjanj, India :
*Diabetes and its Dietetic Treatment.* By B. D. Basu, Major, I.M.S. Tenth Edition. 1919.

From John Wright & Sons. Ltd., Bristol :
*Diseases of the Ear in School Children.* By James Kerr Love, M.D. 1919. Price, 5s. 6d. net.
*The Medical Annual.* 1919. Price £1 net.
*National Health.* By Ferdinand Rees, M.D. 1919. Price 1s. 6d. net.
*On Gun-Shot Injuries to the Blood-Vessels.* By George Henry Makins, G.C.M.G., C.B. 1919. Price 21s. net.

